# Implications of Lingual Orthodontics Compared to Conventional Orthodontics

**DOI:** 10.7759/cureus.72588

**Published:** 2024-10-28

**Authors:** Sneha Nandakumar, Akshay Tandon, Deepak Chandrasekaran, Deenadayalan Purushothaman, Praveen Katepogu, Reshma Mohan, Nidhi Angrish

**Affiliations:** 1 Dental Surgery, SRM Kattankulathur Dental College and Hospital, SRM Institute of Science and Technology, Chennai, IND; 2 Orthodontics and Dentofacial Orthopaedics, SRM Kattankulathur Dental College and Hospital, SRM Institute of Science and Technology, Chennai, IND

**Keywords:** aesthetics, conventional orthodontics, invisible brackets, lingual orthodontics, patient compliance

## Abstract

In orthodontics, the treatment plan and the impact of orthodontic appliances on patients' aesthetic appearance have significantly influenced patients' aesthetic demands over time. Patients considering orthodontic treatment are very concerned about the potential compromise in facial appearance that conventional orthodontic treatment could cause.

Lingual orthodontics offers an aesthetically pleasing appearance compared to conventional orthodontic systems, addressing the growing demand for inconspicuous treatment options. This review examines the implications of lingual orthodontics compared to conventional orthodontics, focusing on aesthetics, oral health outcomes, and patient satisfaction. The benefit of lingual brackets is that when positioned on the teeth's lingual surfaces, they are almost undetectable. However, they are associated with increased difficulty in oral hygiene maintenance due to their placement, leading to higher plaque accumulation that causes compromised periodontal health. Conversely, studies show a lower incidence of white spot lesions on lingual surfaces compared to buccal surfaces, suggesting a reduced risk of caries. Challenges with lingual systems include increased tongue discomfort and speech difficulties. Despite these challenges, lingual orthodontics demonstrates favourable outcomes for patients prioritising aesthetics. Future advancements should focus on improving appliance designs to enhance patient comfort and oral hygiene accessibility, ensuring optimal treatment outcomes. Interdisciplinary collaboration and comprehensive oral hygiene education are essential to maximise the benefits of lingual orthodontics.

## Introduction and background

Malocclusion significantly impairs various aspects of life, including smile, aesthetics, overall appearance, and sociopsychological well-being [1]. Over the past few years, the majority of orthodontic patients have been adults seeking orthodontic therapy, a shift from the previous demography of children. As a result, the number of adults receiving therapy has significantly increased [2]. Due to issues with the appearance of traditional appliances, a large number of individuals who were considering orthodontic treatment declined to proceed [3].

Patients' strong desire for aesthetics has prompted the creation and marketing of a number of aesthetic tools, such as lingual orthodontics, transparent aligners, and attractive brackets [4].

Lingual orthodontics is a specialised and popular branch of orthodontic treatment where braces are positioned on the teeth's lingual surface, making them invisible from the outside. This innovative approach addresses the aesthetic concerns of many patients who desire orthodontic treatment without the visibility of traditional braces [5]. Aesthetic concern is the major factor responsible for the development of lingual appliance systems. The primary motivator for adults is appearance [6]. Lingual orthodontics is the best way to meet patient needs without jeopardising biomechanical efficiency [7].

The brackets in lingual orthodontic treatment are undetachable, which is an attractive feature. The appliance's positioning is the primary distinction between lingual orthodontics and traditional labial orthodontic therapy [8].

In 1726, Pierre Fauchard proposed using appliances on the teeth's lingual surfaces. Pierre Joachim Lefoulon created the first lingual arch appliance in 1841 to help with tooth alignment and expansion [6]. Dr. Craven Kurz first presented the idea of lingual braces in the 1970s, aiming to provide an aesthetic alternative to conventional labial braces [9]. The current lingual treatment began in the mid-1970s, initiated by independent orthodontists in two distinct nations: Dr. Kinya Fujita in Kanagawa, Japan, and Dr. Craven Kurz in the USA, both of whom separately fabricated lingual appliances [6].

At present, lingual orthodontics is a comprehensive system encompassing precise diagnosis, prescribed treatment guidelines, and both laboratory and clinical techniques. The lingual method, however, has had a difficult past. An initial period of enthusiasm followed by a period of rejection and frustration occurred when the lingual approach was first used in a clinical setting [9].

According to a paper by Echarri, we are currently in a period of lingual orthodontic technique renewal. It is now more sophisticated and comfortable, enabling practitioners to achieve results that are on par with the best traditional laboratory techniques [10].

## Review

Methodology

A comprehensive search was undertaken using MEDLINE, PubMed, and Google Scholar to conduct this review. Keywords employed included "orthodontics", "fixed appliances", "lingual orthodontics", "conventional orthodontics", "invisible braces", "oral health", and "dental aesthetics". Owing to the limited availability of research work related to the subject of interest, all existing literature between 1970 and 2024 was included in the review. The search results were improved by reading the bibliographies of the articles that were found. This made sure that the topic of the effects of lingual orthodontics and traditional orthodontics was thoroughly explored.

History of conventional and lingual appliances and brackets

Evolution of Conventional and Lingual Orthodontics

Orthodontics as a formal practice began in the 18th century with Pierre Fauchard, who introduced the concept of using appliances on the teeth, including the lingual surfaces. In the 19th century, Pierre Joachim discovered the first lingual arch for tooth expansion and alignment, and John Farrar described the first lingual appliances [6]. At this time, the primary focus was on functional issues such as correcting malocclusions for better oral health and jaw function, with appliances often bulky and uncomfortable, especially when worn on the labial surfaces of the teeth.

By the early 20th century, advancements in materials and techniques led to the rise of conventional labial orthodontics. Dr. Edward Angle, known as the "father of modern orthodontics", developed the first systematised approach to correcting malocclusion, known as the edgewise appliance, which used metal brackets and wires placed on the labial surfaces of the teeth [11]. This became the foundation of conventional orthodontics. As the field evolved in the mid-20th century, metal brackets became smaller and more efficient, stainless-steel wires improved control of tooth movement, and multibracket systems allowed for the treatment of more complex malocclusions.

In the 1970s, aesthetic demands drove further innovations, leading to the birth of lingual orthodontics. In 1975, Dr. Kinya Fujita of Japan began manufacturing lingual brackets, introducing a lingual multibracket system with a mushroom-shaped arch wire for 3D tooth movement from the lingual surfaces. Around the same time, Dr. Craven Kurz of the USA collaborated with Dr. Jim Mulick to develop a lingual appliance system using plastic brackets on the anterior teeth and metal brackets on the posterior teeth [6]. Despite initial challenges, such as bond failure rates and discomfort, these innovations laid the foundation for modern lingual orthodontics.

The 1980s and 1990s saw significant advancements in both lingual and conventional procedures. Self-ligating brackets that shortened treatment periods, ceramic and plastic brackets with aesthetic appeal, and nickel-titanium wires that produced constant pressures while enhancing patient comfort were all advantages of conventional orthodontics. Digital imaging and 3D printing have enabled the introduction of customised appliances in lingual orthodontics, thereby enhancing comfort and accuracy. Better bonding materials and methods decreased bond failures, while smaller, smoother brackets eliminated pain [9].

Since the 2000s, modern orthodontics has improved labial and lingual treatments. By providing a practically transparent, removable alternative, clear aligners such as Invisalign (Align Technology, San Jose, CA) changed labial systems in traditional orthodontics [12]. Modern technologies, such as 3D imaging and translucent polycrystalline alumina, have improved the treatment's effectiveness and look even further. Furthermore, the development of fully tailored systems such as WIN (DW Lingual Systems GmbH, Bad Essen, Germany) and Incognito (Solventum, Saint Paul, MN) for lingual orthodontics is underway [13]. These systems combine computerised treatment planning and low-profile brackets to provide exact and almost imperceptible outcomes. With thinner, more refined brackets that cause less discomfort, modern lingual systems promote comfort above all else. This makes them a popular option for patients looking for cosmetic solutions without sacrificing the efficacy of their treatment. Although bonding approaches in lingual and labial orthodontics are based on similar concepts, their implementation varies because of differences in bracket design, visibility, and accessibility.

Patient selection

While most malocclusions may be corrected with lingual orthodontics, therapy is more effective in certain circumstances than in others.

Favourable Cases

This treatment technique is most suited for individuals with an anterior deep bite and minor incisor crowding, teeth with long and uniform lingual surfaces free of fillings, crowns, or bridges, and good gingival and periodontal health. This strategy is most suited for keen, conforming patients with a skeletal class I pattern, a mesocephalic or mild to moderate brachycephalic skeletal pattern and patients who can open their mouths and extend their necks properly [7]. 

Unfavourable Cases

Microimplants cannot resolve situations in the dolichocephalic anatomical structure where maximal anchoring is necessary. The lingual tooth surfaces are uneven, short, and worn down. Patients with several crowns, bridges, and extensive repairs often struggle with compliance. Individuals with trismus or restricted mouth openings are unable to extend their necks because of cervical ankylosis or neck injuries [9].

Advantages and disadvantages

Advantages

Lingual appliances are the epitome of aesthetic orthodontics due to their ability to remain discreet and prevent injury to the labial surfaces of the teeth during adhesive removal, debonding, or bonding. In contrast to conventional appliances, the teeth's position can be observed without any obstruction from brackets or arch wires. Deep anterior bites are efficiently corrected using lingual appliances, which intrude anterior teeth and extrude posterior teeth [14].

Disadvantages

The disadvantages of lingual appliances include sore tongues, difficulty speaking, and masticatory problems that persist for more than three months. Lower-profile brackets with custom designs increase comfort and lessen speech problems. Antecedent bite plane effects can hinder mastication, but they typically disappear within one to three months. Lingual surface cleaning is a difficult task that requires patient motivation. Even when they shield the labial surfaces, lingual appliances can still cause gingival oedema and white spot lesions. In situations of anterior crossbite, they lessen bracket loss and successfully handle deep anterior bites. Patients frequently select lingual alternatives because of their cosmetic benefits, despite expenditures [14].

Efficacy and complications

Oral Hygiene and Periodontal Health

Maintaining proper oral hygiene through regular brushing is the most critical part of orthodontic treatment. Proper oral hygiene maintenance will help prevent caries [15]. We should provide the patient with appropriate guidance and encouragement throughout the treatment process [16].

In a study conducted by Vijaykumar et al. [17], they compared two patient groups undergoing conventional orthodontic treatment and lingual orthodontic treatment and found that the lingual group found it more challenging to remove food residue and plaque buildup around the brackets. The analysis of all the indices in the laboratory group revealed that initial gingivitis and plaque formation occurred in the first month. There was a little decline in dental health among patients in the labial group in the third month, although this change was not statistically significant between the two time periods. Compared to the labial group, the lingual group showed greater oral hygiene impairment. Numerous studies indicate that wider lingual brackets reduce the inter-bracket distance [18], complicating dental hygiene practices and increasing the risk of plaque buildup and gingivitis. Hohoff et al. suggested a particular brushing technique to address this problem, and patients with lingual appliances utilise the Waterpik flosser (Water Pik, Inc., Fort Collins, CO) to improve their oral hygiene [19].

Caniklioglu and Oztürk [20] found that the first three-month oral hygiene problem was similar between labial and lingual orthodontics. The study specifically noted that in lingual orthodontics, food impaction was more common. However, between lingual and labial orthodontics, there was no difference in the frequency of bleeding gums or unpleasant taste.

Orthodontic appliances can cause the periodontium to be compromised. In lingual orthodontics, cleaning the lingual surfaces becomes very challenging, leading to the accumulation of plaque that leads to gingivitis. However, using conventional appliances reduces the likelihood of white lesions. A study found that gingivitis is more common in the posterior maxillary teeth when using conventional appliances and in the lingual anterior teeth when using lingual appliances [21]. Another study noted alterations in the microbiota of the periodontium following the bonding of lingual appliances, including an increased incidence of infections such as *Aggregatibacter actinomycetemcomitans* [22].

As a result, teaching oral hygiene is an integral part of every orthodontic treatment. It is important to emphasise the use of adjuncts such as interproximal brushes, sonic electric toothbrushes, fluoride and chlorhexidine mouthwashes, and routine professional cleaning, but the key to success is the patient's dexterity and drive. When planning orthodontic therapy, saliva should be taken into account, as it significantly modifies the cariogenic potential of tooth plaque [23]. Future studies have to concentrate on assessing buffer capacity, salivary flow, and the number of *Streptococcus mutans* throughout a longer length of time.

White Spot Lesions

The major cause of caries in orthodontic patients is plaque buildup between the gingiva and brackets, beneath the arch wire, and around the brackets. According to research, the lingual surface of the teeth has a reduced risk of demineralisation than the buccal surface. van der Veen et al. [24] investigated white spot lesion formation during orthodontic treatment utilising intraoral photos before treatment and qualitative light-induced fluorescence with ocular assessment during therapy. The study discovered that the frequency of white spot lesions on the buccal surface was 4.8 times higher than on the lingual surface and the size of lesions was 10.6 times bigger on the buccal surface.

*Lactobacillus *and* Streptococcus mutans* are the most common organisms responsible for caries; however, in the case of lingual appliances, *Streptococcus mutans* increase in number without a corresponding change in *Lactobacillus* levels [25].

Discomfort

Pain and discomfort are common concerns in lingual orthodontics, with several studies assessing these aspects.

In the study conducted by Wu et al. [26], no change in pain was observed between patients treated with lingual appliances and those treated with conventional appliances over the study period, indicating that the pain levels from both techniques are comparable.

According to Caniklioglu and Oztürk, more tongue soreness was experienced by the lingual appliance patient. This is probably due to the positioning of the lingual brackets, which can irritate and invade the tongue's space, causing pain. In contrast, patients with conventional appliances experienced more lip and cheek pain, likely due to the brackets' proximity to the labial and buccal mucosa. Identifying pain sites associated with different orthodontic treatments is crucial for developing effective pain management strategies, including prevention of discomfort. During the observation, discomfort decreased for both groups; discomfort declined throughout the course of the observation. At one week, both groups reported the highest level of pain, but ratings were lower at one month and three months [20].

A study has noted that pain diminishes over time [27]. It is unclear whether this is due to later treatment stages being less painful or whether patients have adapted to the pain and no longer report it as much. It would be interesting to investigate whether lingual and buccal pain differ in their impact on oral function and whether this affects patients' decision-making in choosing a treatment method.

The investigation consistently noted low use of analgesics, in line with previous research [27]. Interestingly, the first treatment phases, when pain was most intense, saw a higher use of analgesics, confirming the idea that orthodontic treatment causes minimal discomfort [28].

Patients in both groups found it difficult to consume items, especially those that were stiff or fibrous, according to Caniklioglu and Oztürk's research [20]. However, after a month, this problem was resolved for 96.3% of the patients (n = 26) in the laboratory group and 86.2% of the patients (n = 25) in the lingual group. Out of the lingual group, only four patients (13.8%) who had a severe deep bite at the start of therapy still had eating issues after three months. This was due to posterior disocclusion caused by the maxillary anterior lingual brackets' biting planes.

Speech

Speech impediments not only result from physical orthodontic therapy but also have a sociopsychological impact. Labial orthodontics has the least impact on pronunciation. There is a mild speech impairment during the initial days of conventional appliances, which returns to normal after some days [29]. Patients with lingual appliances require at least three months to adapt to the changes, but labial orthodontics needs only two to three weeks for adaptation [30].

Speech difficulties are significantly more common with lingual orthodontic systems than with buccal systems [31]. We draw this conclusion from analyses of various questionnaires and a study that included auditory assessments of speech production with both types of appliances. Evaluations by Caniklioglu and Oztürk [20] and Wu et al. [26] used questionnaires to measure speech difficulties, while another study provided auditory assessments to analyse the impact on speech production [32].

Aesthetic Concerns

Plastic, aesthetically appealing, or tooth-coloured brackets are used to enhance the visual appeal of labial appliances, as appearance is a primary concern. Additionally, multiple-bracket appliances on the lingual surface of the teeth are now available [6].

Over the past few years, orthodontic appliances that are aesthetic have significantly increased in popularity, improving acceptance among patients who are self-conscious with their presentation [33]. As the number of adults seeking orthodontic treatment increases, they prefer aesthetic treatment. As a result, aesthetic orthodontic treatments have become more popular. Unlike labial orthodontic appliances, which are more noticeable to others, lingual orthodontic appliances are much harder to detect [20]. According to Cooper-Kazaz et al. [34], individuals who exhibit narcissistic personality characteristics, obsessive-compulsive behaviour, or somatisation symptoms tend to be early adopters of lingual appliances because of their anxiety. Additionally, research by Bellot-Arcis et al. indicates that patients who opt for lingual appliances often exhibit perfectionist tendencies [35]. The adaptation period for most individuals is similar for both conventional and lingual appliances, approximately 30 days [19], although adaptation is incomplete in some patients with lingual appliances. Despite this, a study has shown that patient satisfaction is comparable between those treated with conventional or lingual appliances, with both groups willing to recommend their respective treatments to others. Custom-made lingual and labial appliances appear to have equal acceptability [36].

Therefore, understanding patients' personality expectations is crucial in determining which type of appliance will best meet their treatment goals and expectations.

## Conclusions

Lingual orthodontics offer a valuable alternative to conventional orthodontics, particularly for patients seeking discreet treatment options. While lingual braces provide significant aesthetic advantages by being nearly invisible, they present unique challenges in terms of application complexity, oral hygiene maintenance, and patient comfort. Despite these challenges, lingual orthodontics has demonstrated lower incidences of white spot lesions compared to conventional systems, indicating potential benefits in reducing caries risk.

Conventional orthodontics remains a reliable and widely accessible option, offering ease of application and shorter treatment durations. However, they can be less favourable for patients prioritising aesthetics. The choice between lingual and conventional orthodontics ultimately depends on individual patient needs, priorities, and orthodontic goals.

Future advancements should focus on improving lingual appliance designs to enhance patient comfort and oral hygiene accessibility. Interdisciplinary collaboration and comprehensive oral hygiene education are crucial to maximising the benefits of both treatment modalities and ensuring optimal outcomes for a diverse range of patients. Ultimately, both lingual and conventional orthodontics play essential roles in contemporary orthodontic practice, offering tailored solutions to meet the diverse needs of patients. 
